# Development of a lab-on-a-chip method for rapid assay of *Xylella fastidiosa* subsp. *pauca* strain CoDiRO

**DOI:** 10.1038/s41598-018-25747-4

**Published:** 2018-05-09

**Authors:** Maria Serena Chiriacò, Andrea Luvisi, Elisabetta Primiceri, Erika Sabella, Luigi De Bellis, Giuseppe Maruccio

**Affiliations:** 1 CNR NANOTEC Institute of Nanotechnology, via Monteroni, Lecce, 73100 Italy; 2 Department of Biological and Environmental Sciences and Technologies, University of Salento, via Monteroni, Lecce, 73100 Italy; 3 Department of Mathematics and Physics, University of Salento, via Monteroni, Lecce, 73100 Italy

## Abstract

*Xylella fastidiosa* subsp. *pauca* strain CoDiRO, a pathogen responsible for Olive Quick Decline Syndrome (OQDS), is strongly threatening the agricultural-based economy of South Italy and making its typical landscape collapse. The bacteria can also infect more than other twenty woody or shrub species and quarantine programs are carried out in Italy. Since symptoms of OQDS like leaf scorching and wilting of canopy may appear several months after infection and some hosts are asymptomatic, a tool for the rapid and early screening of plants is desirable, in order to plan a sudden control strategy and apply programs for pest management. *X. fastidiosa* detection is usually performed by ELISA and PCR methods. In this work, the two standard methods are compared with an innovative on-chip detection strategy for *X. fastidiosa* assay from leaves samples, based on an electrochemical transduction method. The realized lab-on-chip includes also a microfluidic module and its performances are competitive with conventional diagnostic methods in terms of reliability, but with further advantages of portability, low-costs and ease of use. Thus, the proposed technology has the potential to provide a useful assay method for large-scale monitoring programs.

## Introduction

The bacteria *X. fastidiosa* subsp. *pauca* strain CoDiRO was associated to the Olive Quick Decline Syndrome (OQDS or Complesso del Disseccamento Rapido dell’Olivo, CoDiRO), a severe plant diseases that is crushing the production of olive in several districts of Salento (South Apulia, Italy)^1^. The prognosis for infected olive trees is often ill-fated, particularly for the most sensitive cultivar Ogliarola di Lecce and Cellina di Nardò^2,3^. *X. fastidiosa* can infect a broad range of species^4^ and symptoms can be very different. However, a common symptom in many species is the leaf scorching and wilting of canopy. Unfortunately, the first symptoms occurs several months after initial infection (“latent period”), a period of time that may vary according to month since infection, age of the tree and variety^5^. The latent period is a serious threat because it will likely provide enough time for the pathogen to spread far away from the initial point of introduction before being detected. For that reason, large-scale monitoring is desirable.

Several diagnostic protocols were tested for the CoDiRO strain^6^, such as ELISA^7^, PCR^8,9^, direct tissue blot immunoassay^10^ or Loop-mediated isothermal amplification (LAMP)^11,12^. Two real-time PCR protocols are also available^11,13^. Standards for diagnostic protocols for *X. fastidiosa* were recently revised and a flow diagram for the diagnostic procedure for *X. fastidiosa* in plant material was described (European and Mediterranean Plant Protection Organization, EPPO, 2016)^14^. ELISA is a key-test for large-monitoring programs such those carried out in Italy, where more than 150,000 plants were tested in 2016, due to low cost (at least the half) and easier management of large amount of samples compared to molecular tests. For this reason, its inclusion among screening tests is crucial and almost unavoidable. However, analytical sensitivity of ELISA is around 10^4^ CFU/mL, while real-time PCR detects about 10^2^ CFU/mL. Conversely, diagnostic sensitivity and specificity in comparison with naturally infected samples is 100%, the same obtained with molecular tests (EPPO, 2016)^15^. Estimation of workload of laboratory test is also crucial for planning monitoring programs for quarantine pests, whose management is strictly regulated and mainly involve Plant Protection Service and laboratories of Universities or public research centers. Thus, the availability of faster detection methods is desirable.

Among novel approaches, Lab-on-a-Chip (LOC) devices are of increasing interest in many fields of application from clinical diagnostics^16–18^ to agro-food monitoring^19,20^ and are showing potential applications in plant pathology, thanks to low cost, rapidity and sensitivity^21,22^, and may represents an intriguing grower-friendly method for plant pathogen monitoring^23^. Recently, an In-Check system was also tested for quarantine pests such as *Citrus tristeza virus* (CTV), showing as LOC devices may be comparable with capillary electrophoresis single-strand conformation polymorphism (CE-SSCP) analysis and multiple molecular marker (MMM) assays^24^.

In this paper, we report on the validation of a LOC for the specific detection of *X. fastidiosa* subsp. *pauca* strain CoDiRO, in spiked leaf samples, comparing the proposed method to standardized ones (immunoenzimatic and molecular tests). Then a different strain and real samples (naturally infected and healthy) were also analyzed. Our results demonstrate that LOC methods could substantially help in monitor and contrast the spreading of *X. fastidiosa*, by providing a tool with features of low-cost, easy on-field use, and better performance than standard ELISA tests.

## Methods

### Biological samples

Bacteria source (*X. fastidiosa* subsp. *pauca* strain CoDiRO) was obtained from commercial kit (Agritest, Valenzano, Italy) and tests were carried out using dilutions ranging from 10^5^ to 10^2^ CFU mL^−1^. A further bacteria source belonging to a different subspecies (*X. fastidiosa* subsp. *fastidiosa*) was also obtained from commercial kit (Loewe Biochemica, Sauerlach, Germany) and used as control for diagnostic specificity. At least five biological samples were tested for each diagnostic method. Specifically, for ELISA and Lab on chip validation, the dilutions were prepared by artificially adding the inactivated bacterial suspension in fresh homogenized healthy plant tissues, which were then used directly for the assays by testing three replications of each sample. Commercial negative control was used as reference to tests. The same set of samples were used for real-time PCR. To evaluate the sensitivity of the real-time PCR assay, the reaction mix were prepared by adding the proper dilutions of inactivated bacterial suspensions (prepared using Ultrapure DNase/RNase-Free Distilled water, Carlo Erba, Milan, Italy).

Naturally infected or healthy samples were also tested. The plant material was collected from three different symptomatic trees and three different asymptomatic trees. Approximately 1 g of leaf petioles per tree (a pool sample from thirty leaves collected from six branches) was transferred to an extraction bag (BIOREBA, Switzerland) and homogenized using a semi-automatic homogenizer (Homex 6, BIOREBA) at 50% maximum speed, following validated protocols for ELISA/LoC or real-time PCR.

### ELISA and real-time PCR assays

**Figure 1:**
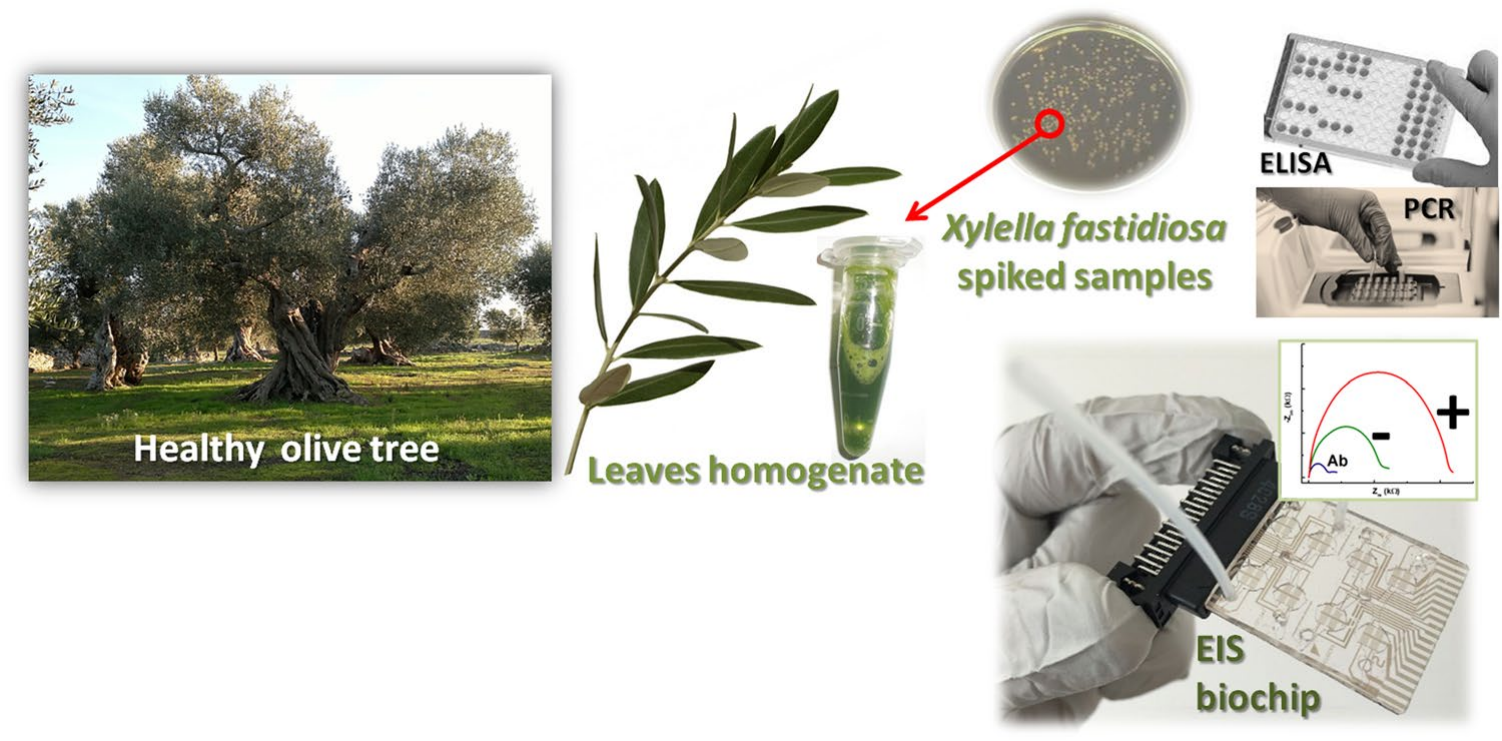
Spiked olive leaves homogenates and real samples (healthy or naturally infected) were tested with two standard techniques and a LOC platform in order to validate the on-chip method for Xylella fastidiosa detection.

To compare performances of LOC, we evaluated samples using ELISA and real-time PCR (Fig. 1). Both tests were carried out following protocols described in EPPO guidelines for *Xylella fastidiosa*^11^.

DAS-ELISA was carried out using recommended antisera and commercial buffers (Agritest). The ELISA kit has been validated for olives, oleander, almond, citrus, oak, grape and other species (i.e. weeds) for detection of *X. fastidiosa* subsp. *fastidiosa*, *multiplex* and *pauca*^7^, showing 100% of diagnostic specificity in comparison with naturally infected samples^14^. No ELISA cross reaction was found against the plant pathogens: *Agrobacerium tumefaciens* biovars 1, 2, *A. vitis*; *Brenneria populi*, *B. quercina*, *B. rubrifaciens*; *Burkholderia andropogonis*; *Clavibcter michiganensis* subsp. *michiganensis*; *Erwinia amylovora*, *Pantoea agglomerans*; *Pantoea stewartii* subsp *stewartii*; *Pseudomonas amygdali*, *P. marginalis* pv. *marginalis*, *P. savastanoi* pv. *savastanoi*, *P. savastanoi* pv *syringe*, *P. savastanoi* pv. *garcae*; *Ralstonia solanacearum*; *Xanthomonas arboricola* pv. *celebensis*, *X*. *arboricola* pv. *corylina*, *X*. *arboricola* pv. *juglandis*, *X*. *arboricola* pv. *pruni*; *Xanthomonas campestris* pv. *citri*, *X. campestris* pv. *populi*, *X. campestris* pv. *vesicatoria*, *X. campestris* pv. *viticola*, *X. hortorum* pv. *pelargonii*^25^. Values of the absorbance at 405 nm (OD_405_) are recorded 4 hours after adding the substrate, using a PerkinElmer 2030 Multilabel reader Victor X5 (PerkinElmer, Wallac OyTurku, Finland). Readings were normalized as R values (OD-sample⁄OD-negative control). R = 2.0 was used as threshold to distinguish a positive response vs. a negative response for tested samples (EPPO, 2010).

Among suggested real-time PCR methods, TaqMan quantitative PCR protocols with XF-F/R primers and XF-P probe^11^ were used. Each reaction was prepared using 5 μL dilution of DNA, 200 nM probe, 400 nM forward and reverse primers, in a total volume of 25 μL. The cycling conditions were: 10 min at 95 °C, followed by 40 cycles of 95 °C for 15 s and 60 °C for 1 min with the final dissociation at 95 °C for 15 s, 60 °C for 30 s and 95 °C for 15 s in a real-time PCR thermal cycler (ABI PRISM 7900 HT Fast Real-Time PCR System, Applied Biosystems). According to EPPO standard^12^, a test was considered positive if it produced an exponential amplification curve. The cycle threshold (C_t_) calculations were carried out using SDS 1.2 software (Applied Biosystems, USA).

### LOC fabrication

**Figure 2:**
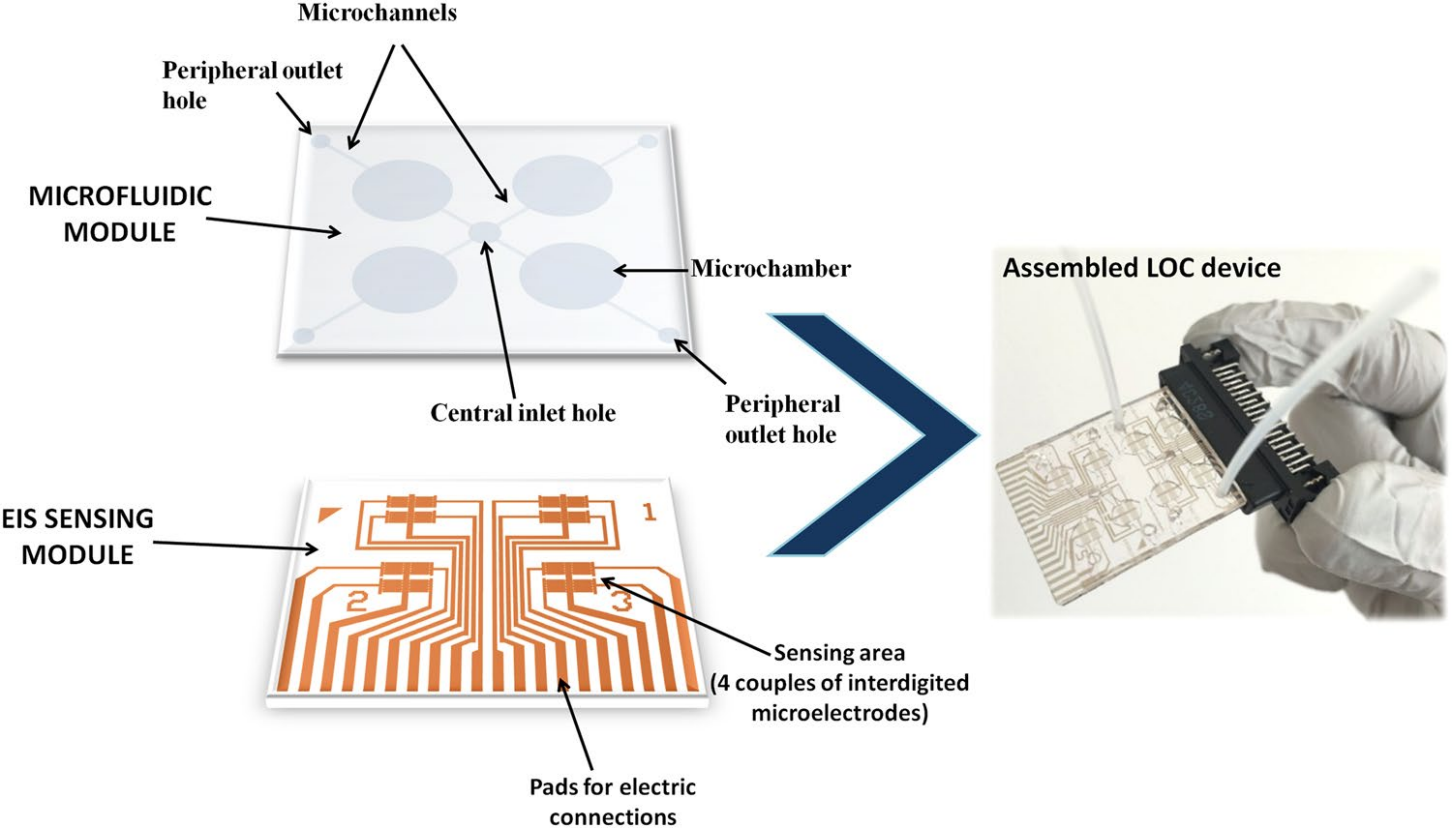
Description of the LOC device for the detection of *Xylella fastidiosa* made up of a sensing and a microfluidic module.

The LOC system used for *X. fastidiosa* detection includes a simple PDMS (Polydimethylsiloxane) microfluidic module providing microchannels and 20 μl microchambers obtained by replica molding. A system of inlet and outlet holes allows the delivery of functionalization solutions and test samples directly on the surface of an array of interdigited metallic microelectrodes realized by optical lithography on a glass substrate. Our current EIS device layout, with a central inlet hole and 4 peripheral outlets per side (half chip), allows the contemporary testing of different samples (Fig. 2). The central inlet was used to perform functionalization steps and to insert the sample to be measured, which is delivered to the four chambers (total volume required for the complete filling of the four microchambers is of around 100 μl). In order to optimize our protocol and measurements, we used the four chambers per side (half chip) for measurements in quadruplicate, but once optimized, each of the four chambers may be used for a separate sample test, considering also that each chamber includes an array of 4 couples of electrodes for redundant measurements (Fig. 2).

Interdigited electrodes (with 10 μm lines and spacing) undergo a functionalization process which allows to specifically bind *X. fastidiosa* cells by means of highly specific antibodies, the same employed for ELISA tests. In particular, the functionalization process includes the overnight deposition of a mixed self-assembled monolayer of beta-mercaptoethanol and mercaptoundecanoic acid (11-MUA) (Sigma Aldrich) able to bind Protein A which in turn contains an Fc antibody-binding specific domain. Antibody used for electrodes functionalization were purchased by Agritest. Passivation of the biosensing layer is achieved by BSA (Bovine Serum Albumin from Sigma Aldrich) 1 mg/ml dissolved in Phosphate Buffered Saline (PBS) pH = 7 for 1 hour at room temperature.

Once the sensing devices have been obtained, calibration experiments with known dilution of bacteria suspensions in PBS were performed, in order to evaluate assay sensitivity. Bacteria dilution from 10^6^ CFU/ml to 10^4^ CFU/ml were delivered to the microfluidic channels and left in static conditions into the microchambers for 1 hour to allow the biorecognition between antibodies immobilized on the electrodes’ surface and *Xylella fastidiosa* cells suspended. Subsequently 1 ml of washing PBS solution was delivered into the system and chambers were filled with a redox couple solution of hexacyanoferrate_(II/III)_ K_3_[Fe(CN)_6_]/K_4_[Fe(CN)_6_] (1:1) at a concentration of 10 mM in order to perform electrochemical impedance measurements. An Autolab PGSTAT30 was employed to acquire impedance spectroscopy data by applying a sinusoidal 15 mV AC voltage in a range of frequencies from 10^5^ Hz to 0.1 Hz. Beyond spiked samples, the LOC response was also tested for the recognition of the phytopathogen in leaf matrix, in order to evaluate the influence of a more complex real sample. To this aim, a preliminary rough filtration of the leaf homogenate was achieved by means of a sterile gauze piece double folded, in order to retain larger leaves debris which may adhere to the electrodes giving false positive results. After filtration, aliquots of leaves homogenates were spiked with serial dilution of *X. fastidiosa* suspensions.

### Data availability

All data generated or analysed during this study are included in this article after statistical analysis in the form of mean values with standard deviation. Impedance spectroscopy raw data were exported as ASCII files and were processed using OriginPro 2016.

## Results

### ELISA and real-time PCR assays

In ELISA tests, R of positive control was more than 5, while absorbance of negative control was below 0.2. Positive readings were obtained up to a dilution of 10^4^ CFU mL^−1^ (Table 1). In real-time PCR, amplification curve of positive control was exponential (C_t_ 19.8), while negative control gave no amplification during 40 cycles. Samples were considered as positive to test up to the dilution of 10^2^ CFU mL^−1^. No differences in both ELISA and real-time PCR results were observed with the subsp. *fastidiosa* control.

**Table 1:** Test results (+ = positive, − = negative) of samples ranging from 10^5^ to 10^2^ CFU mL^−1^, according to ELISA test or real-time PCR.

Sample dilution	CFU mL^−1^	ELISA		Real-time PCR	
		R	Result	C_t_	Result
1:1	1.0 * 10^5^	5.33	+	19.8	+
1:5	2.0 * 10^4^	4.22	+	20.4	+
1:10	1.0 * 10^4^	2.16	+	21.3	+
1:20	5.0 * 10^3^	1.35	−	22.2	+
1:50	2.0 * 10^3^	0.99	−	23.6	+
1:75	1.3 * 10^3^	0.99	−	24.2	+
1:100	1.0 * 10^3^	1.00	−	24.6	+
1:1000	1.0 * 10^2^	0.92	−	28.9	+
Control	0	1.00	−	NA	−

R = OD-sample/OD-negative control; C_t_ = cycle threshold. The reported values were obtained from the average of two technical replicates for each sample.

In ELISA tests on naturally infected samples, R was set at 4.77 ± 1.23; in real-time PCR test, mean C_t_ was 20.67 ± 1.04. Samples collected from asymptomatic trees showed R value around 1.00 and gave no amplification during 40 cycles of real time PCR. These samples were also used for LoC tests.

### LOC detection

Electrochemical impedance biochips were realized as an array of metallic interdigited microelectrodes with lines and spaces of 10 μm. Sensing areas have been aligned with a microfluidic module including microchannels and microchambers in which biological reactions have been performed. Impedance spectra are shown below in the form of Nyquist plots where the *x* axis represents the real component of impedance Z_re_ and on the *y* axis the complex component -Z_im_ is plotted. The reported impedance curves have been obtained by at least three repeated measurements and error bars represent the standard deviations. A Randles circuit can be used to model impedance spectra and estimate electron transfer resistance (R_et_, roughly the intercept with *x* axis) which is influenced by electrode modifications and increases consequently to the deposition of molecular layers and analytes, during both functionalization or detection phases.

**Figure 3:**
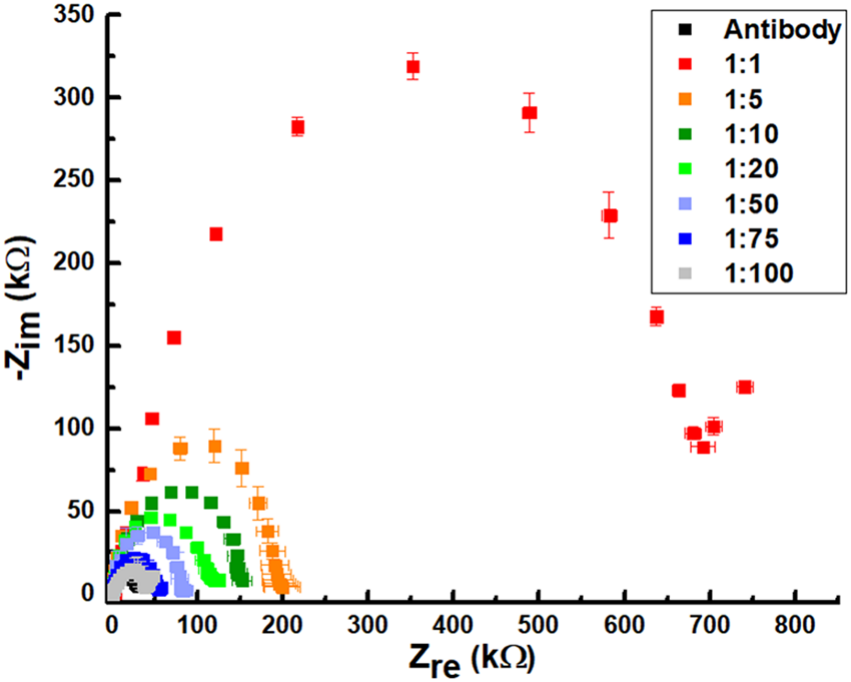
Nyquist spectra exemplifying the device response to serial dilution of stock solution (1:1) containing 10^5^ CFU/ml of *Xylella fastidiosa* in PBS.

**Figure 4:**
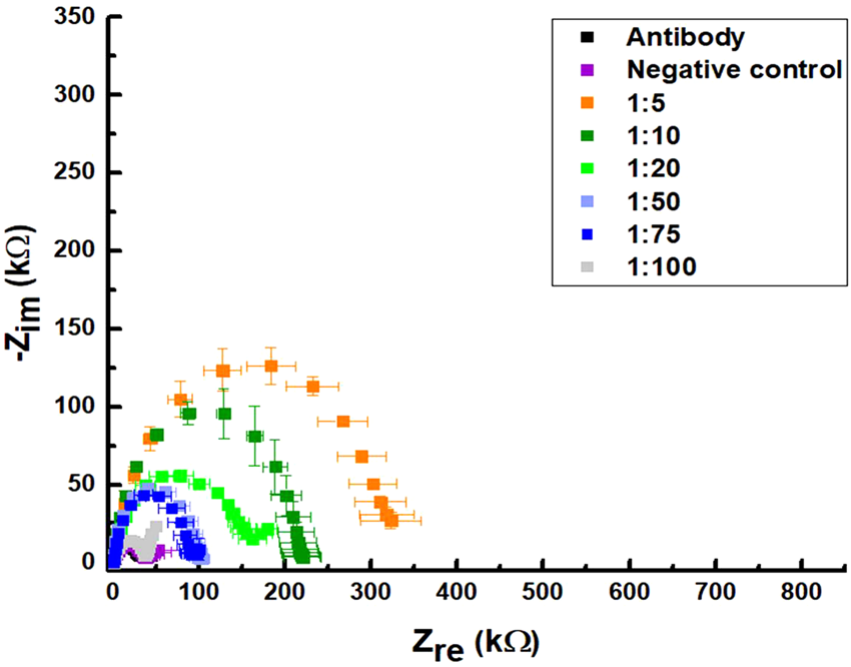
Nyquist spectra of spiked samples in healthy olive leaves homogenate. Serial dilutions of samples have been obtained from stock solution containing 10^5^ CFU/ml.

As a first step, the device was measured after functionalization phase, in order to identify the impedance contribution related to the antibodies anti- *X. fastidiosa*. In this case, a reproducible impedance value of around 31 kΩ was obtained (black squares in Figs 3 and 4). Successively, a calibration using serial dilutions of *Xylella fastidiosa* was performed, resuspending aliquots of a 10^5^ CFU/ml suspension in PBS. A remarkable variation of impedance values has been observed when resuspended *Xylella fastidiosa* cells were incubated in the microfluidic chambers of the device, thus providing a robust system able to monitor the biorecognition events of antigens at the interface between the functionalized electrodes and the solution. In particular, stock solution resulted in a value of R_et_ of around 705 kΩ (red squares in Fig. 3) with an increment in impedance with respect to the baseline of around 675 kΩ; ratios tested inside the microfluidic and sensing platform include 1:10 dilution of the stock solution (which resulted in R_et_ around 151 kΩ), 1:20 (R_et_ around 121 kΩ), 1:50 (R_et_ around 84 kΩ), 1:75 (R_et_ around 58 kΩ). The curve associated with 1:100 ratio (R_et_ around 35 kΩ corresponding to 10^3^ CFU/ml), still separated, is quite overlapped with antibody baseline (cyan squares in Fig. 3). Impedance spectroscopy is thus very effective for monitoring biorecognition events on surface-modified electrodes.

As a first step to demonstrate ability to detect *X. fastidiosa* from real vegetable samples, we tested our functionalized platform with olive leaf homogenates, obtained by mechanical mashing and minimally pre-treated by filtration through a sterile gauze. Matrix contribution to impedance spectroscopy over the antibody layer was evaluated as around 10 kΩ, resulting in a R_et_ around 40 kΩ, corresponding to the grey squares (negative control) in Fig. 3, probably related to an unspecific absorption of matrix molecular components over the microelectrode surface. In order to obtain a calibration curve with real samples and to establish the matrix effect also with increasing concentrations of *X. fastidiosa*, we enriched healthy control leaf homogenates to obtain spiked samples with a concentration from 10^3^ to 2 × 10^4^ CFU/ml by using the same bacteria suspension employed for calibration experiments in PBS. Leaf samples spiked with 10^3^ CFU/ml, corresponding to a 1:100 ratio of dilution, resulted in R_et_ around 40 kΩ. This value is again overlapped with negative control of healthy leaf homogenate over the antibody layer, thus providing a lower limit for sensitivity of the proposed platform in case of real samples.

**Figure 5:**
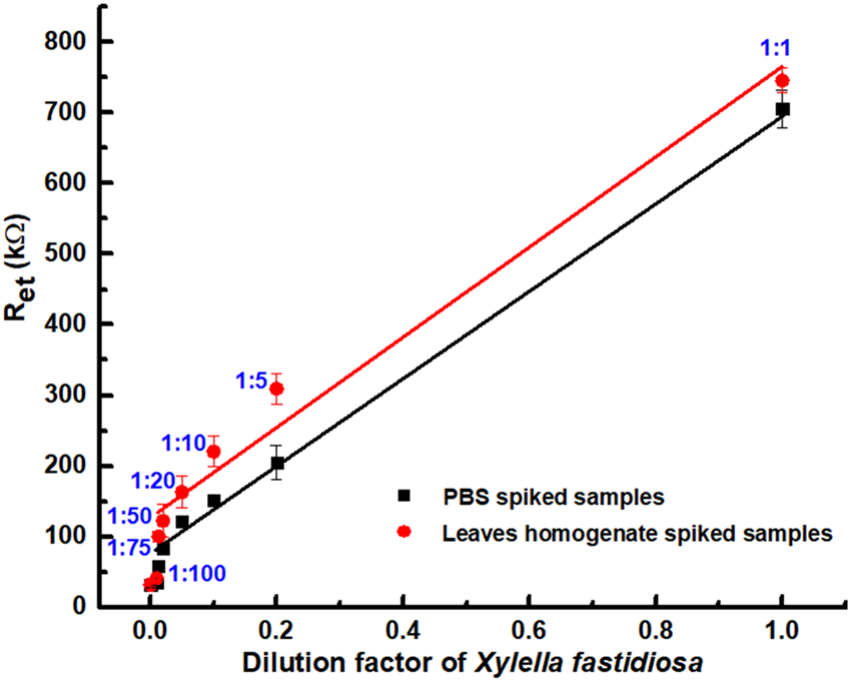
Calibration curves for *Xylella fastidiosa* in a EIS device. Black line is related to serial dilution of stock solution on PBS; red line is related to spiked leaves homogenate samples.

For both calibrations in PBS and in spiked leaves samples, a response curve has been obtained and these trends are reported in Fig. 5 (black and red dots respectively), showing detection limits of 1 × 10^3^ corresponding to the 1:100 dilution of stock solution for PBS spiked samples, and 1:75 for homogenate leaves sample. Data points may be fitted with a linear behaviour for highest concentrations (while the slope becomes higher at low concentrations). In particular for PBS spiked samples (black dots and line in Fig. 5), an almost linear response has been obtained down to 1:75 sample, while for leaves homogenate spiked samples (red dots and line) this is possible down to 1:50 dilution. This may be probably due to a unspecific absorption of matrix components (such as plant wall debris) which interfere with a more sensitive detection of *X. fastidiosa* cells.

The applicability of our optimized EIS test on samples coming from naturally infected (or healthy) trees, was also demonstrated. Specifically, we performed the analysis on leaves homogenates coming from asymptomatic and symptomatic trees, treated with the same protocol for spiked samples and carrying the analysis in parallel with ELISA and PCR methods.

**Figure 6:**
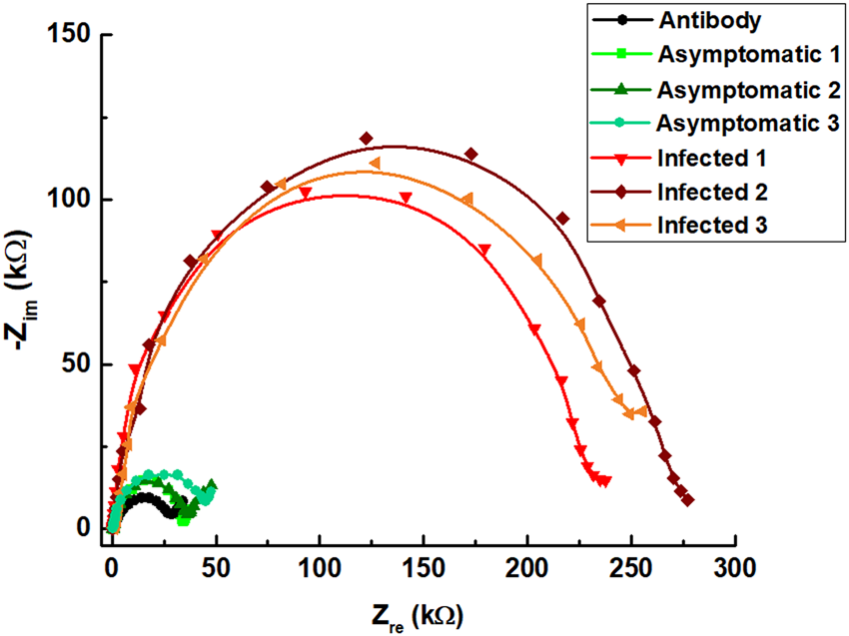
EIS spectra obtained by testing asymptomatic and infected trees with LOC device.

From these tests we found that asymptomatic trees reported impedance values (green lines in Fig. 6) clearly shifted toward the antibody baseline (black line around 30 kΩ), while EIS analysis of infected trees resulted in increased impedance values in a range from 225 to 275 kΩ.

## Discussion

Conventional diagnostic techniques are frequently time consuming and laborious. Immunological-based detection methods, such as ELISA, can be automated to be more time effective and job-saving but they have some limitations: low sensitivity and false negative results. New technologies are sought to overcome these critical points and to provide portable and miniaturized devices that can be used directly in the field by giving rapid response to tests. In this work, we set up a reliable, low cost and label-free assay based on Electrochemical Impedance Spectroscopy able to detect 1.3 × 10^3^ CFU/ml of *X. fastidiosa* cells from spiked samples of olive leaves homogenates. We also performed ELISA and PCR tests in order to compare results and to validate our innovative assay. In Table 2, the results in terms of sensitivity of the three methods are summarized. These findings place our developed assay in a competitive position because of a better sensitivity with respect to ELISA methods. ELISA tests fail in detecting microorganism at dilution factors higher than 1:10, while our LOC platform is able to identify pathogens’ cells down to 1:75 dilutions, showing a limit of detection 7.5 times better than ELISA.

**Table 2:** Results from analysis carried out on leaves homogenate spiked with known quantity of *Xylella fastidiosa*.

Sample dilution	ELISA	PCR	LOC
1:1	**+**	**+**	**+**
1:5	**+**	**+**	**+**
1:10	**+**	**+**	**+**
1:20	**−**	**+**	**+**
1:50	**−**	**+**	**+**
1:75	**−**	**+**	**+**
1:100	**−**	**+**	**−**
1:1000	**−**	**+**	**−**

All tests were performed blindly in parallel with ELISA, PCR and LOC methods. A comparison of results in terms of sensitivity reveals that LOC is more effective to detect *X. fastidiosa* with respect to ELISA technique, while PCR reach the best sensitivity.

As far as PCR-based methods are concerned, they exhibit better performances compared to LOC devices with positive results even at very high dilutions (down to 1:1000). However, nucleic acid-based methods are expensive and require advanced equipment and qualified professionals to be performed. In this respect, our device can represent a good compromise among the need for sensitivity and assay accessibility, showing intermediate performances between ELISA and PCR. We also tested LOC devices for confirming the presence/absence of *X. fastidiosa* in naturally infected olive trees and asymptomatic trees, and our results were also confirmed by standard methods assays.

Moreover, the proposed LOC platform is suitable to be improved in order to include a user-friendly sample preparation method by the integration of microfluidic tools for on chip sample processing such as filtration for debris removal^23^. Another competitive aspect of the proposed LOC devices concerns the costs, which were estimated as 1.8 or 6 time lower than ELISA and real-time PCR respectively. Costs of LOC devices (around 5 €/device) are mainly due to the glass substrate and the metallization. A significant saving with respect to standard methods is instead related to a minimal employ of antibodies since functionalization of EIS devices requires 1 µl/ml antibody concentration. The high suitability for downscaling of the number of sensing areas in each device will not affect volumes of reagent required for functionalization neither the cost of a single device, but will improve the number of tests which may be simultaneously performed. All these aspects, together with the limited dimension of the platform (few square centimeters) and the possibility to easily integrate the electronic connections into portable devices like laptops or smartphones, makes our optimized biochip suitable for on-field use. Thus, this technology paves the way to monitoring and screening olive trees directly where epidemic threats are expected or where symptoms of the disease have not been already pointed out, in order to apply timeless and necessary control actions. A further benefit of our developed assay is the possibility to set up multiplexed tests by including also other phytopathogens for early identification of different plant pests. However, even if validated antibodies are used for LOC fabrication, inter-laboratory tests are also needed in future to definitely prove robustness of this technique.
